# How to make methodological decisions when inferring social networks

**DOI:** 10.1002/ece3.6568

**Published:** 2020-08-07

**Authors:** André C. Ferreira, Rita Covas, Liliana R. Silva, Sandra C. Esteves, Inês F. Duarte, Rita Fortuna, Franck Theron, Claire Doutrelant, Damien R. Farine

**Affiliations:** ^1^ Centre d’Ecologie Fonctionnelle et Evolutive Univ Montpellier CNRS EPHE, IRD Univ Paul‐Valery Montpellier 3 Montpellier France; ^2^ CIBIO‐InBio Research Centre in Biodiversity and Genetic Resources Vairão Portugal; ^3^ Department of Collective Behavior Max Planck Institute of Animal Behavior Konstanz Germany; ^4^ FitzPatrick Institute of African Ornithology DST‐NRF Centre of Excellence University of Cape Town Rondebosch South Africa; ^5^ Department of Biology University of Konstanz Konstanz Germany; ^6^ Centre for the Advanced Study of Collective Behaviour University of Konstanz Konstanz Germany

**Keywords:** assortativity, group living, methods, *Philetairus socius*, social behavior, social network analysis

## Abstract

Social network analyses allow studying the processes underlying the associations between individuals and the consequences of those associations. Constructing and analyzing social networks can be challenging, especially when designing new studies as researchers are confronted with decisions about how to collect data and construct networks, and the answers are not always straightforward. The current lack of guidance on building a social network for a new study system might lead researchers to try several different methods and risk generating false results arising from multiple hypotheses testing. Here, we suggest an approach for making decisions when starting social network research in a new study system that avoids the pitfall of multiple hypotheses testing. We argue that best edge definition for a network is a decision that can be made using a priori knowledge about the species and that is independent from the hypotheses that the network will ultimately be used to evaluate. We illustrate this approach with a study conducted on a colonial cooperatively breeding bird, the sociable weaver. We first identified two ways of collecting data using different numbers of feeders and three ways to define associations among birds. We then evaluated which combination of data collection and association definition maximized (a) the assortment of individuals into previously known “breeding groups” (birds that contribute toward the same nest and maintain cohesion when foraging) and (b) socially differentiated relationships (more strong and weak relationships than expected by chance). This evaluation of different methods based on a priori knowledge of the study species can be implemented in a diverse array of study systems and makes the case for using existing, biologically meaningful knowledge about a system to help navigate the myriad of methodological decisions about data collection and network inference.

## Graphical abstract

Determining how to build social networks for new study systems can be challenging. Pilot studies and a priori knowledge can facilitate making methodological decisions. We provide an empirical example of how to make methodological decisions by comparing different foraging networks in a colonial bird.

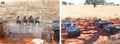

## 1 INTRODUCTION

Social network analysis (SNA) has gained popularity in behavior ecology as a tool to study the processes underlying the associations between individuals and the consequences of those associations (Cantor et al., 2019). It allows biologists to characterize not only the social environment experienced by a single individual in the population, but also the broader social characteristics of a population (Newman, 2010). However, while the methods involved in analyzing a network are reasonably well‐explained (e.g., Whitehead, 2008), there are many decisions involved with the design of data collection and creating the network itself (Farine & Whitehead, 2015).

Decisions about the design of a study can have consequences on the inferred network structure (James, Croft, & Krause, 2009). How can we know that our design decisions produce a suitable network for the species and the type of hypotheses we are studying? There is generally little discussion of the considerations made when designing a network‐based study, with most published papers presenting their design as a “fait accompli.”

When analyzing a social network, the key decision that needs to be made is how to define the relationships (edges) connecting the individuals (nodes). This definition can include two main components. The first is the set of considerations relating to how data are collected (e.g., direct observations versus. video recordings), and the second is the decisions that relate to how observations are turned into edge weights (e.g., rate of interactions versus. time spent together). In most systems, the scope of decisions about data collection appears constrained by methodological limitations, but often there are choices that reflect some trade‐offs. For example, is it better to collect fewer data across more individuals at once or to collect more detailed data on fewer individuals? These decisions in turn have consequences for hypotheses testing. Davis, Crofoot, and Farine (2018) provided a useful general discussion on the impact of these trade‐offs. However, there is no general guidance on how to quantify the relative value of different approaches when faced with designing methods for real data collection.

Once data are collected, the second set of considerations that arise reflect decisions about how to calculate the strength of the relationships among individuals. While one aspect determining the accuracy of a network to ensure that sufficient data are collected (see Farine & Strandburg‐Peshkin, 2015), how data are used to generate quantitative measures of connection strength (edge weights) can also have a large impact on the resulting network. For example, different association indices (Cairns & Schwager, 1987; Hoppitt & Farine, 2018) or different types of data resolution (e.g., the number of grooming bouts versus the amount of time spent grooming) can be used to estimate the strength of a given relationship.

The lack of guidance on how to evaluate different approaches to data collection and network inference might lead researchers to try several different methods and to select the one that best correlates with the predictions of the study (e.g., a positive relationship between a given network metric and survival). Such a correlation could give a false impression that the method chosen produces a network that is successfully capturing the species' or population social structure. At worse, this approach could constitute a multiple hypotheses testing scenario, elevating rates of type I errors because the design decisions are made based on producing a result. This risk is elevated when combined with opportunities to calculate multiple network metrics (e.g., degree and betweenness). For example, a researcher might be interested in understanding whether specific individual attributes, such as personality, correlate with one or multiple network centrality metrics (e.g., Aplin et al., 2013; Boogert, Farine, & Spencer, 2014; Chock, Wey, Ebensperger, & Hayes, 2017; Johnson et al., 2017; Moyers, Adelman, Farine, Moore, & Hawley, 2018; Wilson, Krause, Dingemanse, & Krause, 2013). In the absence of significant results, it could be tempting to change a posteriori the methods by which the network *is generated* from the data, such as changing the time window or the proximity criterion used to consider that two individuals are associated. While such an example is extreme, there is an important challenge arising from not knowing whether failing to reject a given null hypothesis is a consequence of the expected pattern not being present or the researchers' failure to correctly construct the network. We therefore need an approach that avoids creating circularity, that is, using the same data tested in different ways to corroborate a given hypothesis, as well as using the significant result to corroborate the quality of the information contained in the network. This problem is exacerbated by the lack of information, in most published studies, about how design decisions were made, that is, whether they were made arbitrarily (or based on a published study), based on pilot studies, or if explored in the way described above (but see Boogert, Farine, et al., 2014; Castles et al., 2014; Mourier, Bass, Guttridge, Day, & Brown, 2017, for some exceptions).

Two complementary approaches can help with making decisions about the design of a network study. The first is to collect pilot data, testing different data collection setups (e.g., varying the number of simultaneous observers collecting data). Unfortunately, this is often not possible, not done, or not reported. The second is to run exploratory a priori analyses aimed at comparing different competing networks resulting from different network generation methods and in networks with different edge definition. For both approaches, we propose (and show) here that comparison of the different methods is made possible by testing and interpreting simple hypotheses that we generally consider a network from that study species should support, *before* testing the hypothesis of central interest.

Capturing structure in a given species' network that aligns with a priori knowledge on the species can be interpreted as an approximation of hypothetical ground‐truthed network (which is something that is unlikely to be available when working with nonhuman animals). For example, in a species where mother and offspring or breeding pairs create strong social bonds, we expect that the implemented method would result in a network that would be able to capture these preferred associations (i.e., estimate the edge weight within a family/breeding pair as being significantly greater than those between other sets of individuals, see Boogert, Farine, et al., 2014 and Hobson, Avery, & Wright, 2014). Such an analysis would then provide information about whether a network is capable of differentiating, and therefore capturing, one or more important aspects of the biology of the system.

In this paper, we provide an empirical example of how to make decisions about the design of a network study using exploratory a priori test. We start by formulating simple tests of hypotheses to help guide the design of data collection and network inference. We conducted this study in a population of a colonial and cooperatively breeding bird, the sociable weaver (*Philetairus socius*). In this population, individuals are marked with PIT tags allowing automatic data collection at feeders containing supplemental food. We decided to collect associations in a feeding context not only because this has been shown to be important and meaningful in other bird studies (e.g., Aplin, Farine, et al., 2015), but also as a result of the general insights on the social foraging behavior of this species that have been reported in previous studies on this population (Lloyd, Altwegg, Doutrelant, & Covas, 2017; Rat, van Dijk, Covas, & Doutrelant, 2015; Silva et al., 2018). Therefore, it seems reasonable to assume that information about social relationships within a colony could be obtained from foraging associations (see Farine, 2015), if the study is well designed.

We evaluate the performance of different study designs at extracting two fundamental structural aspects of the social system in our study species (herein our test statistics). The first metric is social differentiation, which we calculate using the coefficient of variation (CV). Because sociable weavers' colonies are large, we do not expect birds to have the same relationship strength with all colony members (i.e., low values of CV). Thus, an informative network should be one that features large differences in the connection strengths that individuals have in their social network (i.e., having many small and large values, rather than many intermediate values). However, solely relying on social differentiation can be misleading as high values can be obtained as a result of nonsocial factors (e.g., low sampling or spatially distributed individuals), nor should maximizing social differentiation necessarily result in the most biologically accurate network. Thus, our second metric for testing if the edges in the foraging network reflect social bonds is one that aims to capture something more specific about sociable weaver biology, assortment by breeding group. Sociable weaver colonies contain several breeding groups composed of breeders with their helpers (usually a breeding pairs plus one to four helpers; Covas, Dalecky, Caizergues, & Doutrelant, 2006). Assortment is a measure of the tendency for connections in a network to be more common among similar than among dissimilar types of nodes (Farine, 2014; Newman, 2003). Thus, assortment by breeding group is a metric that would capture the tendency of individuals from the same breeding group to be more strongly connected to one another in the network. We expect this because while aggression between individuals at food patches is common (sociable weavers typically forage in large groups containing many colony members), aggression between members of the same breeding groups is rare (suggesting higher tolerance for other breeding group members, Rat, 2015). Thus, we expect members of the same breeding group to be disproportionately detected together, resulting in a real social network that is assorted by breeding group membership.

First, we quantify the effects of data collection decisions on the resulting values of social differentiation and assortment by breeding group. Specifically, we test how allowing different numbers of individuals to feed simultaneously impacts our two test statistics. As data collection decisions are challenging to make when starting a new study, but are critical because they can have a major impact on the robustness of the resulting network(s) (i.e., the network, are sufficient to reliably estimate properties of the real social structure; Davis et al., 2018). In our case, it is not clear whether sociable weavers with stronger social relationships feed more synchronously across repeated foraging visits than birds with weaker relationships, or whether the differences in behavior are better defined as the patterns of foraging within a foraging visit (i.e., with who within the flock the individuals prefer to associate in close proximity). The former requires more widespread effort (i.e., determining only the foraging flock composition), while the latter requires more refined data to be collected within foraging flocks (i.e., more opportunities to record individuals simultaneously at the same site). These two approaches represent a clear cost trade‐off as the former can be achieved with fewer resources compared to the latter. For example, when collecting data using RFID technology, having one feeder fitted with an RFID antenna can be enough to obtain the identity of all individuals in a foraging flock (e.g., Jones, Evans, & Morand‐Ferron, 2019), multiple RFID antennas working simultaneously are needed to determine if two birds present in the same flock feed in close proximity. We therefore compare different setups for collecting associations that differ in the number of birds that can be detected in an automated RFID system at the same time.

Second, we focus on how to define associations from within a given dataset. Specifically, we compare three different approaches to generate quantitative measures of edge weights in the network, and test how these subsequently impact our test statistics. Two approaches are based on number of co‐occurrences in “foraging events.” These are akin to using the “gambit‐of‐the‐group” approach (Franks, Ruxton, & James, 2010; Whitehead & Dufault, 1999), where all birds that are detected (i.e., observed) in a flock together are considered to be associated. However, this approach discards more detailed data that could be available about within‐flock structure and instead assumes that birds with strong relationships will tend to be co‐observed in the same flock more often than those with weak relationships. The third approach is a more direct measure of the proportion of time that two individuals spend in close proximity within the flocks. That is, because we collected data at multiple readers in close proximity, we could estimate how much time two individuals spent on neighboring feeders.

Our aim is to provide guidance on how to make decisions when dealing with choices in the design of data collection and/or network inference. We achieve this by drawing from an empirical example in which we use existing knowledge of our study species guide decisions for designing a network study. In doing so, our study highlights how relatively simple approaches, using short periods of pilot data collection and evaluating network data against basic knowledge about the study species, can facilitate making methodological decisions that could have long‐term impact on the success of a study. While our focus is on collecting and analyzing network data, such an approach goes beyond studies of animal social networks.

## 2 METHODS

### 2.1 Study scope and model species

We studied a population of sociable weavers at Benfontein Nature Reserve, situated ca. 6 km southeast of Kimberley, in the Northern Cape Province, South Africa. The sociable weaver is endemic to the semiarid savannahs of southern Africa (Maclean, 1973a) and feeds mainly on insects and seeds (Maclean, 1973c). Sociable weavers build large nests, usually on *Acacia* (*Vachellia*) trees, with several independent chambers where the birds roost throughout the year and where breeding takes place (Maclean, 1973b). This species exhibits three noticeable cooperative behaviors: building the communal nest, feeding nestlings of others, and communal nest defense from predators such as snakes (e.g., Boomslang, *Dyspholidus typus* and Cape cobra, *Naja nivea*). The size of a colony can range from less than ten to several hundred individuals. The breeding pairs can either breed with or without helpers (30%–80% of breeding attempts have helpers; Covas, Du Plessis, & Doutrelant, 2008).

This study is part of a long‐term research program which involves the annual capture of 14 colonies to maintain an individually marked population (all individuals are marked with a unique metal ring and color combination: Covas et al., 2008; Paquet, Doutrelant, Hatchwell, Spottiswoode, & Covas, 2015). At five colonies, all birds are also marked with a passive integrated transponder (PIT tag, enclosed in a plastic leg ring). These colonies ranged in size from 43 to 82 individuals (colony size estimated from the annual captures in September 2017).

### 2.2 Breeding groups' identification

Breeding groups were determined using video recordings of the chambers during the reproductive season of October 2017 to January 2018. We routinely inspected all colonies every 3 days to identify initiation of new clutches. We visited chambers in the days around the expected hatching date to determine the age of the nestlings and then recorded each breeding group for at least 2 hr when the chicks were between 8 and 20 days old. We considered an individual as part of the group if it was seen feeding the chicks at least 3 times, as occasionally some individuals try to feed but are expelled by the breeding group.

### 2.3 SNA data collection

During December 2017 and April 2018, we collected two rounds of association data in a feeding context using artificial feeders at the 5 PIT‐tagged colonies. For all the 5 colonies, the feeding location was 80–205 m away from the colony.

**FIGURE 1:**
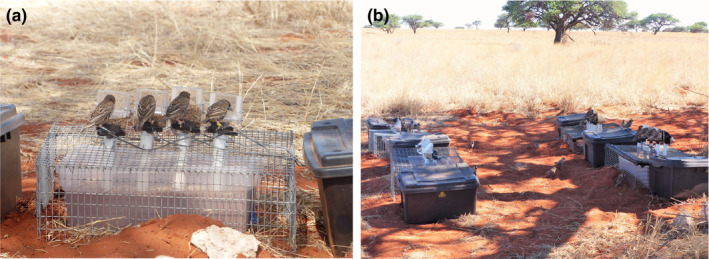
Setup for collecting associations (a) A feeding box with birds feeding at the four plastic feeders and the RFID antennas (b) the low competition setup with four feeding boxes. Photographs by Cecile Vansteenberghe

Data from three of the five colonies were collected using a setup containing 2 feeding boxes (high competition setup), each with 4 perches and 4 small standard plastic bird feeders. Each small feeder allowed for only one bird to feed at a time and was fitted with a RIFD antenna (Priority1rfid, Melbourne, Australia) connected to a data logger (Figure 1a). Data from these three colonies were collected for 14 days (sampled continuously).

At two of the five colonies (of similar sizes, 43 and 44 individuals), we evaluated alternative methods for collecting feeding association by varying the number of birds that could feed at the same time. We introduced an alternative setup comprising 4 feeding boxes instead of 2 (low competition setup; Figure 1b), allowing birds to spread out more when visiting the feeding station and, therefore, for us to collect more observations of cofeeding. Data for each setup (high and low competition) were collected within the same study period, alternating between the setup each day. This design allowed us to make direct comparisons of the two setups without a cofounding factor of time period in which the data were collected, the number of days that each setup was used to collect data, or which colony data were collected from. We collected 10 days of data for each setup.

### 2.4 Edge weight calculations

The stream of data collected in the field comprised of temporal sequences of PIT tag codes detected at each of the feeder perches. From these data, we calculated associations from our observation data in two different ways:

1. Co‐occurrence method. We first used the gambit of the group, where all individuals that are observed together are considered to be equally connected to each other (i.e., a flock) and the strength of connections is estimated based on the repeated patterns of co‐occurrences of individuals in the same observation. However, there are several ways a flock can be defined (see Farine & Whitehead, 2015). Here, we used an established method of inferring flocks based on the time differences between two detections. The start and end times of a “wave” of individuals considered to be forming a flock are determined by a Gaussian mixture model (GMM; using R package “asnipe” Farine, 2013; following Psorakis et al., 2015), which is an automated clustering algorithm designed to detect peaks, or clusters of detections, in the temporal profile of activities at the artificial feeders. This approach uses data from the feeding behavior of the entire set of individuals as part of determining the associations between any two individuals.
2. Time overlap method. We estimated association strengths directly from the data by calculating the total time that two individuals overlapped while feeding at the same feeding box. This approach does not use any data from other individuals when determining the associations between two individuals.

**FIGURE 2:**
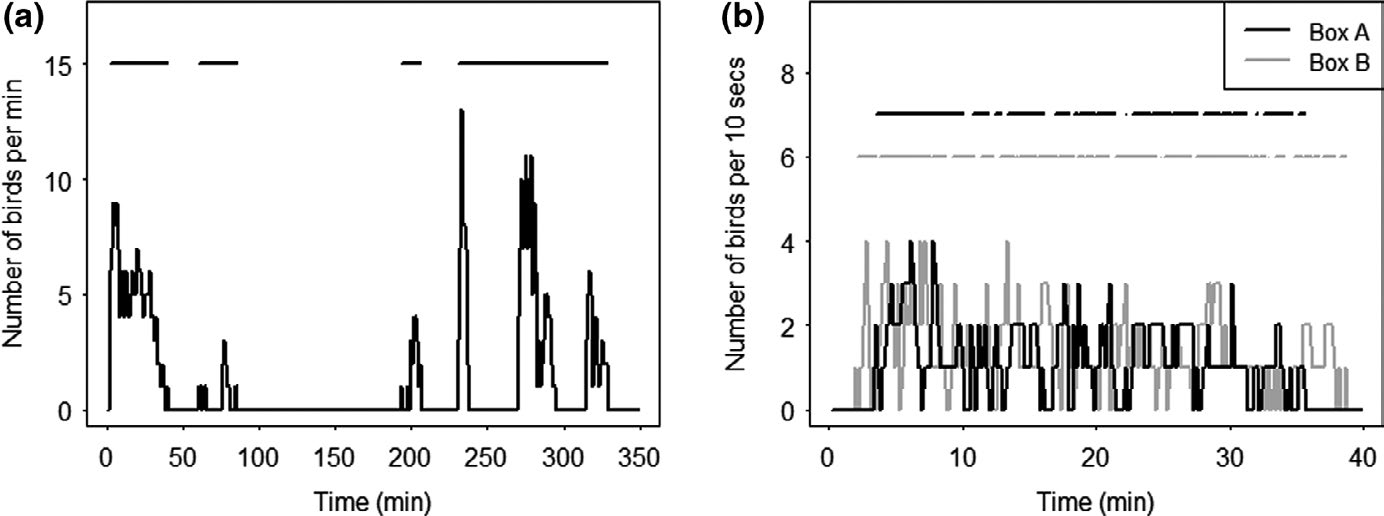
Example of applying the GMM algorithm method. (a) Sociable weaver visits to a feeding location during one morning. The top straight lines represent the foraging events resulting from the first GMM. (b) The foraging events resulting from the second GMM, discriminating between the two feeding boxes and using only visits from the first event determined by the first GMM (corresponding to the first horizontal line segment on Figure 2a)

**FIGURE 3:**
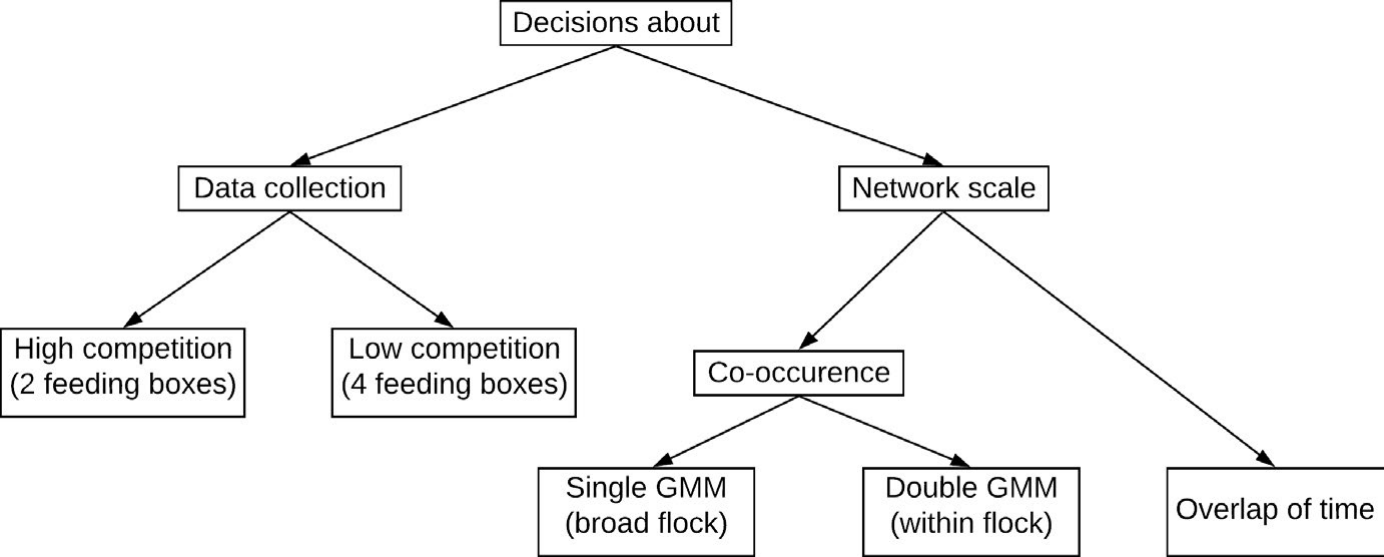
Flow diagram illustrating the steps for the two different comparisons of the study: comparing different methods for calculating edge weights and comparing different data collection setups

These two methods are described in more detail below. For the co‐occurrence method, we used two variants (see Figure 2): one focused on the association at the broad flock level (single GMM) and the other added a second step of estimating association within each flock (double GMM). Therefore, three different network types were compared for each combination of colony (see Figure 3 for an illustration of the different comparisons done in this work).

#### 2.4.1 Co‐occurrence networks

*Single GMM (broad flock):* We built networks using the rates of co‐occurrence on the same so‐called “foraging events” as commonly done in other studies (e.g., Aplin et al., 2013). Foraging events were defined using a single run of the GMM (single GMM network) directly on the raw daily RFID feeder data, which splits the temporal data in different foraging events based on peaks of activity on the feeding boxes for that day (following Psorakis et al., 2015). We considered each feeding box as a different location to allow us to split the flock spatially in order to archive a greater resolution in detecting preferred associations. We inferred the association strengths (edge weights) among colony members from their copresence across all foraging events. We used the simple ratio index: the number of times that two individuals were in the same foraging event divided by the number of foraging events that contained at least one of the two individuals.

*Double GMM (within flock):* Since our study species is colonial and highly gregarious, we believed that to differentiate the relationships among colony members we would need edges based on co‐occurrences at a finer scale than what has traditionally been used for other species (i.e.,. using the single GMM). Therefore, we used the Gaussian mixture model approach to define associations among individuals using a two‐step procedure. Because the data from the feeders are quite discontinuous in this population (i.e., all individuals tend to visit foraging patches together and then all depart together in a very synchronized manner), we first detected the broader activity profile at the set of feeder boxes. We did this by grouping the individuals' detections across all feeder boxes at a location in a given day into 1 min blocks and used the GMM to extract the arrival and departure times of broad foraging events (see Figure 2a). After this first step, we used the GMM again, but this time to detect waves of activity within each of the foraging events determined by the first GMM run. In the second run, we considered each feeder box (containing 4 RFID perches each) as a different location and used detections at a 1‐s resolution. Considering each feeding box as a different location allowed us to split the data on the flock spatially, while running the GMMs within each foraging event allowed us to decrease the time scale and forced the GMM to split into shorter feeding bouts (Figure 2b), thereby allowing the detection of within‐flock spatial and social preferences. We inferred the association strengths among colony members from their copresence across all feeding bouts generated from the second runs of the GMM (double GMM network). As with the single GMM approach, we used the simple ratio index.

#### 2.4.2 Time overlap networks

For the time overlap networks, we directly calculated the proportion of total feeding time during which two individuals were feeding simultaneously in the same feeding box (i.e., the time that birds spent feeding side‐by‐side). Here, edges were calculated by taking the sum of time that two individuals spent feeding at the same time at the feeding box divided by the sum of the total time that at least one of these two individuals were present at the feeder (which is also the simple ratio index, but more explicitly time‐based rather than occurrence‐based). This method aimed to define a stricter scale at which we consider that two individuals were associated and represents the degree of tolerance to feed together. This method can be more relevant for colonial and very gregarious species such as sociable weavers, since all members of the colony are often found foraging together and are already connected by colony membership, and since our interest is to find a sublevel of sociality within this colony structure.

### 2.5 Hypothesis testing

We evaluated each network we produced by testing if they were significantly different from networks generated from randomizations of our data and if they generated patterns that reflect a biologically meaningful social aspect of this species. Specifically, we evaluated the utility of each network we generated (3 variants times 2 data collection methods) according to two test statistics:

1. The coefficient of variation in edge weights, to test which method would result in more differentiated networks. Low CV values represent a network in which individuals are equally connected, whereas a high CV value means that there are both strong and weak relationships detected. We do not expect sociable weavers to associate equally with all members of their colony, but they should have equal opportunity to associate with all others (i.e., they are co‐occurring in the same space). Thus, CV is a suitable test statistic of general patterns of social differentiation in our species.
2. The weighted assortment coefficient (following Farine, 2014) using breeding group membership as the individual trait. High values of assortment coefficients represent disproportionately strong associations among individuals with the same trait (here between members of the same breeding group), while low values represent no such structure. Thus, our second statistic is a more explicit test of an a priori hypothesis about who individuals should be connected to in the network. Because not all individuals of the colony could be attributed to a breeding group, since not all breeding pairs managed to successfully reproduce during this breeding season, we restricted the network to the subset of individuals known to belong to a breeding group.

We tested the statistical significance of the CV and the assortment coefficients by comparing the test statistics calculated from the observed networks with the same statistics calculated from 1,000 random networks generated using permutations of the observed data (see Farine, 2017). For the co‐occurrence method, we generated random networks following the method first described by Bejder, Fletcher, and Brager (1998), using the R package asnipe (Farine, 2013). Briefly, for the single GMM networks, we selected pairs of observations of individuals from different foraging events and then swapped these individuals. For the double GMM network, the approach is similar; however, pairs of observations of individuals were selected from the same foraging events (from the first run of the GMM) and at the same feeder, but from different feeding bouts (from the second run of the GMM). For the overlaps of time networks, we split the observed data by the foraging events defined by the first run of the GMM in the double GMM method and swapped the identity of the individuals within each foraging event. That is, we performed restricted node permutations (following Aplin, Firth, et al., 2015, but restricted by time and space, rather than by space only). By randomizing individuals' detections events within each foraging event, we aimed to keep constant, as much as possible, other factors besides social preferences that might contribute to the structure of the network (such as variation in individuals' propensities to join flocks visiting feeders).

For all the 5 colonies, we compared the CV and the assortment coefficients from the 3 different types of networks (singles GMM, double GMM, and overlap of time). Additionally, for 2 of those 5 colonies, we also compared each of the network types resulting from data collected using high and low competition setups. This allowed us to test whether we could improve our networks not just in terms of edge definition but also regarding the design of data collection by changing the number of birds that can access food simultaneously. As illustrated in the diagram of Figure 3, the decisions about our method for constructing a suitable network for the sociable weavers were guided by both the setup design and the edge definition. Addressing these two questions might appear to be a sequential scheme, that is, first looking at feeder saturation and after deciding if there was or not a significant improvement in using the 4 feeding boxes, addressing the scale problem (by comparing the different types of networks) or the other way (first the scale and then the feeder saturation). However, we did not address this as a sequential problem, since the two types of comparisons (comparisons of scale and comparisons of feeder saturation) are not easy to disentangle. In order to compare the high competition with the low competition setup, we need a reliable edge definition which can only be obtained by comparing the 3 types of networks. However, the best edge definition might differ when using different methods for collecting data.

## 3 RESULTS

We found that our methodological approach for evaluating different methods for data collection and network inference yielded informative results that could be directly applied when making decisions about study design. All of the methods we used generated networks that were significantly different from random. From an edge definition perspective, the overlap of time method consistently generated networks with higher CV (Table 1) and higher values of assortment (Table 2). While the co‐occurrence methods were able to detect the predicted positive assortment by breeding group in most colonies, the overlap of time method consistently produced considerably higher assortment coefficients. The single GMM co‐occurrence method was able to generate well‐differentiated networks, but performed worse with the assortment coefficients being closer to zero (Table 2). These results suggest that the networks produced by the overlap of time method performed better at capturing a sublevel of sociality within the colony that we expected to be captured in a network of sociable weaver with an appropriate edge definition.

**TABLE 1:** Comparison between the CVs of the three different types of networks obtained using a setup with two and four feeding boxes

Network type	Colony ID	Detected individuals	Two feeding boxes		Four feeding boxes	
			CV	*p*	CV	*p*
Co‐occurrence single GMM	11	34	0.548	<.001	0.414	<.001
	20	27	0.516	<.001	0.556	<.001
	27	38	0.738	.026	–	–
	43	27	0.646	.002	–	–
	71	59	0.608	.02	–	–
Co‐occurrence double GMM	11	34	0.646	.004	0.804	<.001
	20	27	0.530	<.001	0.752	<.001
	27	38	0.877	<.001	–	–
	43	27	0.770	<.001	–	–
	71	59	0.700	.05	–	–
Overlap of time	11	34	2.143	<.001	2.500	<.001
	20	27	1.414	<.001	1.872	<.001
	27	38	1.770	<.001	–	–
	43	27	1.351	<.001	–	–
	71	59	1.731	<.001	–	–

Note

Number of individuals per colony: colony 11:34; colony 20:27; colony 27:38; colony 43:27; colony 71:59.

**TABLE 2:** Comparison between the assortment by breeding groups for the three different types of networks obtained using a setup with two and four feeding boxes

Network type	Colony ID	Individuals in groups	Number of groups	Two feeding boxes		Four feeding boxes	
				Assortment (SE)	*p*	Assortment (SE)	*p*
Co‐occurrence single GMM	11	20	8	−0.005 (0.026)	<.001	−0.020 (0.027)	<.001
	20	10	3	−0.063 (0.086)	.14	0.053 (0.094)	<.001
	27	20	6	−0.017 (0.028)	.002	–	–
	43	17	5	−0.013 (0.013)	<.001	–	–
	71	19	4	−0.005 (0.039)	.018	–	–
Co‐occurrence double GMM	11	20	8	0.018 (0.029)	.004	0.092 (0.045)	<.001
	20	10	3	−0.022 (0.088)	.12	0.232 (0.105)	<.001
	27	20	6	0.009 (0.032)	.052	–	–
	43	17	5	0.049 (0.041)	.012	–	–
	71	19	4	0.012 (0.042)	.002	–	–
Overlap of time	11	20	8	0.297 (0.074)	<.001	0.389 (0.088)	<.001
	20	10	3	0.160 (0.175)	<.001	0.637 (0.087)	<.001
	27	20	6	0.141 (0.066)	<.001	–	–
	43	17	5	0.094 (0.055)	<.001	–	–
	71	19	4	0.190 (0.070)	<.001	–	–

Note

Number of individuals (number of groups) per colony: colony 11:19 (6); colony 20:10 (3); colony 27:20 (6); colony 43:17 (5); colony 71:19 (4).

From a data collection methods perspective, using four boxes instead of two resulted in higher CVs and in higher assortment coefficients in both colonies (Tables 1 and 2). In other words, using more feeding boxes at a given site resulted in greater power to discriminate between same breeding group associations within a colony across all the three types of networks. This effect was more pronounced in the co‐occurrence method than in the overlap of time method.

Together these results show that using more feeders and an edge definition based on overlap of time produced networks that are able to capture the expected assortment by breeding group and performed better than other methods. We can now use this method to construct networks to test our hypotheses of interest in future research such as testing if specific individual attributes (e.g., personality traits) influence social relationships among the individuals.

## 4 DISCUSSION

Using our empirical example, we have shown how knowledge about the study population can be used to help making decisions about the data collection design and determining how to calculate the strengths of social relationships. We have also shown that, as expected, different edge definition and experimental designs in the same context can result in different networks: some presenting a low coefficient of variation and thus a network in which individuals are more equally connected, and others with a higher CV, and thus a network containing a higher number of both stronger and weaker relationships. Importantly, we found that the methods that appear best suited to our study system differ from those that have been widely used in studies of PIT‐tagged songbird populations, highlighting the need to ensure that methods are tailored to the specific systems under investigation.

In the case of the sociable weaver, we showed that using the time that individuals spent together, rather than data on simpler co‐occurrences, generates networks that best captured network features that we a priori identified as being important. For example, the assortment coefficients by breeding group were more than ten times higher in the time‐based networks than in the networks generated from co‐occurrences. While using a more time resolved co‐occurrence method (double GMM) resulted in a better network to capture assortment by breeding group relative to the standard GMM method, it still performed worse than a network based on the time that individuals spend in close proximity. This would be expected for a species such as the sociable weaver, in which colonies can forages in flocks always containing the same individuals. Thus, while we found that a network definition based on the overlap in time provided the networks that best captured a priori knowledge of the study species' social structure (i.e., the breeding group), it might not necessarily be the best method for all questions or study systems. For example, tits (*Paridae*) spend the winter in flocks with highly dynamic membership with membership changing over the course of minutes (Farine et al., 2015) and pairs of blue tits (*Cyanistes caeruleus*) can be detected forming through their increased comembership in the same flocks (Beck, Farine, & Kempenaers, 2020). Thus, using a single GMM can extract the social signal from tit flocks because this signal is contained in broader patterns of flocking rather than fine‐scale patterns of social proximity. Hence, for each study system, and for each purpose, researchers should carefully consider what is the best way to construct their networks, potentially requiring experimenting while avoiding trying the different methods on a given hypotheses of interest.

We also generated new insights into how to design data collection protocols. For the sociable weaver, we found that networks generated using more sampling opportunities (in this case a higher number of feeder boxes available simultaneously) produced networks with higher assortment by breeding unit. Our finding is in line with the suggestions made in a recent methodological paper that simultaneous sampling data can result in more robust networks (Davis et al., 2018). Even though our analyses are based on only two colonies, the reason for this improvement is easy to explain. Having fewer feeders available increases competition for access to feeders, which, in turn, might reduce the ability for groups of preferred associates within a colony to forage at the same time, and force them to forage with less preferred conspecifics. Alternatively, competition for access to the resource could go as far as causing only the more dominant individuals of each group to have access to the resource, meaning that we would fail to sample subordinates. In either case, having fewer feeders means that birds could not clearly express the social preferences we would expect them to have in more dispersed and more natural resources.

In social network studies, the number of individuals that can be detected at the same time (or in a given time window) is rarely considered or reported. In our study, 8 or 16 individuals could be detected simultaneously, contrasting with studies on tits and other songbirds that use feeders which typically detect one (Jones et al., 2019) or two (e.g., Aplin, Farine, et al., 2015; Beck et al., 2020) birds simultaneously. Other field studies, such as recent work on wild zebra finches (*Taeniopygia guttata*) (Brandl, Farine, Funghi, Schuett, & Griffith, 2019) used feeders with a restricted entrance allowing multiple flock members to enter and exit feeders together. Reporting the proportion of birds detected feeding together could allow assessing whether restricting data collection to fewer simultaneous observations dilutes true social bonds, causing lower network resolution and potentially leading to less accurate associations, as it appears to be the case in the sociable weaver. This issue becomes an important consideration for studies with limited budgets or researcher time as field studies often face the trade‐off between maximizing replication across individuals (i.e., sampling more individuals in total) versus maximizing the precision of the data collected (i.e., sampling individuals simultaneously). In our study, one setup requires twice as much equipment, meaning that we could only sample at half the locations or revisit each location half as often. Simulation studies suggest that collecting more simultaneous data is generally preferable (Davis et al., 2018), because networks require many replicated observations of each possible pair of individuals in order to be robust (see Farine & Strandburg‐Peshkin, 2015). Such improvement in the resulting networks might well justify the additional economic cost associated with having more feeders or having technology capable of detecting multiple individuals in close proximity.

Our study also illustrates how different data collection methods and algorithms for estimating association strengths can generate different networks (see also Castles et al., 2014). While the different networks that are collected may be correlated (see Farine, 2015), this does not mean they are all equally powerful at testing a hypothesis. However, when testing network quality, the choice of which a priori knowledge to use is also critical. For instance, while a method that was guided using the assumption that individuals prefer to associate with other members of the same breeding group might be appropriate to study phenomena that potentially involve a social preference (e.g., testing if individuals assort by their propensity to cooperate), it might not be feasible to study phenomena where casual or random interactions play an important role such as the spread of contagious disease. For example, in the European starlings (*Sturnus vulgaris*), the spread of a novel foraging task in a social group was predicted by a perching network but not by a foraging network, possibly as a result of a perching network better capturing social preferences than a foraging network in a captive setting (Boogert, Nightingale, Hoppitt, & Laland, 2014), while Hoyt et al. (2018) tested multiple ways of characterizing social connections among individuals but these failed to map on to the observed spread of an experimentally introduced pathogen mimic (UVF dust). Advanced analytical techniques can also help to discriminate which network is the most informative at prediction the spread of information. For example, Firth, Sheldon, and Farine (2016) found that the social network collected after experimentally segregating flocks of tits better predicted the discovery of new resources than the social network collected prior to the experimental manipulation. Such techniques could form the basis for pilot studies aimed at investigating how best to map the global structure of wild populations.

Previous studies used simulation‐based approaches (Bonnell & Vilette, 2020; Psorakis et al., 2015) to identify the best method to discriminate patterns of social connections, or video data to confirm that the detection data match reality (Evans, Devost, Jones, & Morand‐Ferron, 2018; Nomano, Browning, Nakagawa, Griffith, & Russell, 2014). Here, we demonstrated that using a priori knowledge about the study species or population can be helpful in making decisions about which network to use—which we believe is a stronger approach as collecting pilot data captures many of the nuances that come with collecting field data. Anticipating the potential limitations of the method used for data collection provide researchers with the opportunity to make the necessary adjustments before collecting the actual data, avoiding revisiting their methods and even hypotheses a posteriori. The crucial point to keep in mind, however, is that researchers should aim to make a priori decisions (even if some are inevitably arbitrary) about methods for collecting data and building networks and ensuring that these are independent of any later tests of hypotheses. Failing to do so would decrease the rates of type I errors in social network studies. Researchers could also make use of preregistration services (Nosek, Ebersole, DeHaven, & Mellor, 2018) to publish the research questions, discuss different methods, plan analyses and pilot studies before collecting the data and observing the research outcomes. This would not only greatly improve the credibility of research findings but it would be also useful information to other researchers that are planning their studies.

We have tried to draw attention to the decisions that underlie social network analyses. Many recent papers provide guidance on how to construct networks (reviewed in Farine & Whitehead, 2015). However, to our knowledge, little guidance is available about how to make system‐specific decisions about data collection (e.g., number of individuals detected simultaneously) that can be critical to the results obtained. We show that integrating existing knowledge about the species' social behavior in making decisions can be a simple and very powerful way of informing which approach is the best one. The concepts we present, involving forming and using simple hypothesis testing to evaluate competing networks and help guide the process of building a network, are easily generalized to other system. They go beyond breeding group membership (which is specific to cooperative breeders), bird studies, foraging associations, RFID setups, or questions of co‐occurrence versus time overlap, which we merely used here as empirical examples to illustrate the advantages of the proposed approach. Any set of networks can be compared with a relevant biological metric, regardless of the methods used. For example, when studying a group of primates using direct observations one has to decide for how many hours per day to observe each group and each individual, how many observers to hire to collect the data (similar to our question of how many feeders to use), or even choose between different sampling approaches (Altmann, 1974). As the comparison of co‐occurrence versus the overlap of time done here, decisions on how to define the edges of the network also have to be made: are edges defined by spatial proximity more meaningful for a given species and for specific question of interest than edges defined by other social interactions? These decisions are easier to make if we know what patterns to expect in a social network of for a given study species. Basing methodological decisions on tests of a priori known biological properties of the study system, ideally while simultaneously collecting pilot data, will result in more robust network data than copying studies from other systems. This should also avoid the pitfalls of combining exploration of network inference with testing new hypotheses.

In this paper, we provide a structured approach that can be used to make design decisions in network, or other, studies. In addition, we also call for researchers to provide more information about the rationale leading to their decisions. In our case, we took advantage of the information obtained as a result of a long‐term project on a cooperatively breeding species, which provided information on composition of breeding groups. In other projects, this type of information might not be available or relevant, but other types of information, such as the importance of mated pairs which are expected to share strong social bonds (see Beck et al., 2020; Boogert, Farine, et al., 2014; Brandl et al., 2019; Firth, Voelkl, Farine, & Sheldon, 2015) could be used. Further, we reiterate that our study clearly highlights the need for data collection and analysis methods to be tailored to each study system, as different approaches (all of which are valid and exist in the literature) can produce quite different outcomes. We hope that once sufficient studies report their design process, as we have here, we will be able to identify some general guidelines for animal social network data collection and analysis.

## CONFLICT OF INTEREST

None declared.

## AUTHOR CONTRIBUTIONS

**André C. Ferreira:** Conceptualization (equal); data curation (equal); formal analysis (equal); methodology (equal); writing–original draft (equal). **Rita Covas:** Funding acquisition (equal); resources (equal); supervision (supporting); writing–original draft (equal); writing–review and editing (equal). **Liliana R. Silva:** Data curation (equal); methodology (equal); writing–review and editing (equal). **Sandra C. Esteves:** Data curation (equal); methodology (equal); writing–review and editing (equal). **Inês F. Duarte:** Data curation (equal); writing–review and editing (equal). **Rita Fortuna:** Data curation (equal); writing–review and editing (equal). **Franck Theron:** Data curation (equal); writing–review and editing (equal). **Claire Doutrelant:** Funding acquisition (equal); methodology (equal); resources (equal); supervision (supporting); writing–original draft (equal); writing–review and editing (equal). **Damien R. Farine:** Conceptualization (equal); methodology (equal); supervision (lead); writing–original draft (equal); writing–review and editing (equal).

## ETHICAL APPROVAL

All experiments were conducted under permission of the Northern Cape Department of Tourism, Environment and Conservation (permit FAUNA1338/2017), and under the approval of the Ethics Committee of the University of Cape Town (2014/V2/RC). The procedures implemented in this work involved the capture, confinement, ringing, handling, and blood sampling of adult birds and nestlings. All potential invasive procedures were conducted by experienced ringers. Adult birds were not kept for more than 3 hr and were released in small groups. Any bird showing signs of fatigue were kept in an aviary and released upon recovery.

## Data Availability

Code and data for reproducing the entire contents of this article are available at Dryad Digital Repository https://doi.org/10.5061/dryad.p8cz8w9mx

## References

[ece36568-bib-0001] Altmann, J. (1974). Observational study of behavior: Sampling methods. Behaviour, 49(3–4), 227–266. 10.1163/156853974X00534 4597405

[ece36568-bib-0002] Aplin, L. M. , Farine, D. R. , Morand‐Ferron, J. , Cockburn, A. , Thornton, A. , & Sheldon, B. C. (2015). Experimentally induced innovations lead to persistent culture via conformity in wild birds. Nature, 518, 538–541. 10.1038/nature13998 25470065 PMC4344839

[ece36568-bib-0003] Aplin, L. M. , Farine, D. R. , Morand‐Ferron, J. , Cole, E. F. , Cockburn, A. , & Sheldon, B. C. (2013). Individual personalities predict social behaviour in wild networks of great tits (*Parus major*). Ecology Letters, 16, 1365–1372. 24047530 10.1111/ele.12181

[ece36568-bib-0004] Aplin, L. M. , Firth, J. A. , Farine, D. R. , Voelkl, B. , Crates, R. A. , Culina, A. , … Sheldon, B. C. (2015). Consistent individual differences in the social phenotypes of wild great tits, *Parus major* . Animal Behavior, 108, 117–127. 10.1016/j.anbehav.2015.07.016 PMC4579410 26512142

[ece36568-bib-0005] Beck, K. B. , Farine, D. R. , & Kempenaers, B. (2020). Winter associations predict social and extra‐pair mating patterns in a wild songbird. Proceedings of the Royal Society B, 287(1921), 20192606 10.1098/rspb.2019.2606 32070248 PMC7062020

[ece36568-bib-0006] Bejder, L. , Fletcher, D. , & Brager, S. (1998). A method for testing association patterns of social animals. Animal Behavior, 56, 719–725. 10.1006/anbe.1998.0802 9784222

[ece36568-bib-0007] Bonnell, T. R. , & Vilette, C. (2020) Constructing and analysing time‐aggregated networks: The role of bootstrapping, permutation and simulation. Methods in Ecology and Evolution, 1–13. 10.1111/2041-210X.13351 [Epub ahead of print].

[ece36568-bib-0008] Boogert, N. J. , Farine, D. R. , & Spencer, K. A. (2014). Developmental stress predicts social network position. Biology Letters, 10, 20140561 10.1098/rsbl.2014.0561 25354917 PMC4272205

[ece36568-bib-0009] Boogert, N. J. , Nightingale, G. F. , Hoppitt, W. , & Laland, K. N. (2014). Perching but not foraging networks predict the spread of novel foraging skills in starlings. Behavioural Processes, 109, 135–144. 10.1016/j.beproc.2014.08.016 25178191

[ece36568-bib-0010] Brandl, H. B. , Farine, D. R. , Funghi, C. , Schuett, W. , & Griffith, S. C. (2019). Early‐life social environment predicts social network position in wild zebra finches. Proceedings of the Royal Society B, 286(1897), 20182579. 30963840 10.1098/rspb.2018.2579 PMC6408881

[ece36568-bib-0011] Cairns, S. J. , & Schwager, S. J. (1987). A comparison of association indexes. Animal Behavior, 35, 1454–1469.

[ece36568-bib-0012] Cantor, M. , Maldonado‐Chaparro, A. , Beck, K. , Carter, G. , He, P. , Hillemann, F. , … Farine, D. R. (2019). Animal social networks: Revealing the causes and implications of social structure in ecology and evolution. EcoEvoRxiv.

[ece36568-bib-0013] Castles, M. , Heinsohn, R. , Marshall, H. H. , Lee, A. E. G. , Cowlishaw, G. , & Carter, A. J. (2014). Social networks created with different techniques are not comparable. Animal Behavior, 96, 59–67. 10.1016/j.anbehav.2014.07.023

[ece36568-bib-0014] Chock, R. Y. , Wey, T. W. , Ebensperger, L. A. , & Hayes, L. D. (2017). Evidence for a behavioural syndrome and negative social assortment by exploratory personality in the communally nesting rodent, *Octodon degus* . Behaviour, 154, 541–562. 10.1163/1568539X-00003433

[ece36568-bib-0015] Covas, R. , Dalecky, A. , Caizergues, A. , & Doutrelant, C. (2006). Kin associations and direct vs indirect fitness benefits in colonial cooperatively breeding sociable weavers *Philetairus socius* . Behavioral Ecology and Sociobiology, 60, 323–331. 10.1007/s00265-006-0168-2

[ece36568-bib-0016] Covas, R. , Du Plessis, M. A. , & Doutrelant, C. (2008). Helpers in colonial cooperatively breeding sociable weavers *Philetairus socius* contribute to buffer the effects of adverse breeding conditions. Behavioral Ecology and Sociobiology, 63, 103–112. 10.1007/s00265-008-0640-2

[ece36568-bib-0017] Davis, G. H. , Crofoot, M. C. , & Farine, D. R. (2018). Estimating the robustness and uncertainty of animal social networks using different observational methods. Animal Behavior, 141, 29–44. 10.1016/j.anbehav.2018.04.012

[ece36568-bib-0018] Evans, J. C. , Devost, I. , Jones, T. B. , & Morand‐Ferron, J. (2018). Inferring dominance interactions from automatically recorded temporal data. Ethology, 124(3), 188–195. 10.1111/eth.12720

[ece36568-bib-0019] Farine, D. R. (2013). Animal social network inference and permutations for ecologists in R using asnipe. Methods in Ecology and Evolution, 4, 1187–1194.

[ece36568-bib-0020] Farine, D. R. (2014). Measuring phenotypic assortment in animal social networks: Weighted associations are more robust than binary edges. Animal Behavior, 89, 141–153. 10.1016/j.anbehav.2014.01.001

[ece36568-bib-0021] Farine, D. R. (2015). Proximity as a proxy for interactions: Issues of scale in social network analysis. Animal Behavior, 104, e1–e5. 10.1016/j.anbehav.2014.11.019

[ece36568-bib-0022] Farine, D. R. (2017). A guide to null models for animal social network analysis. Methods in Ecology and Evolution, 8, 1309–1320. 10.1111/2041-210X.12772 29104749 PMC5656331

[ece36568-bib-0023] Farine, D. R. , Firth, J. A. , Aplin, L. M. , Crates, R. A. , Culina, A. , Garroway, C. J. , … Sheldon, B. C. (2015). The role of social and ecological processes in structuring animal populations: A case study from automated tracking of wild birds. Royal Society Open Science, 2(4), 150057 10.1098/rsos.150057 26064644 PMC4448873

[ece36568-bib-0025] Farine, D. R. , & Strandburg‐Peshkin, A. (2015). Estimating uncertainty and reliability of social network data using Bayesian inference. Royal Society Open Science, 2, 150367 10.1098/rsos.150367 26473059 PMC4593693

[ece36568-bib-0026] Farine, D. R. , & Whitehead, H. (2015). Constructing, conducting and interpreting animal social network analysis. Journal of Animal Ecology, 84, 1144–1163. 10.1111/1365-2656.12418 26172345 PMC4973823

[ece36568-bib-0027] Firth, J. A. , Sheldon, B. C. , & Farine, D. R. (2016). Pathways of information transmission among wild songbirds follow experimentally imposed changes in social foraging structure. Biology Letters, 12(6), 20160144 10.1098/rsbl.2016.0144 27247439 PMC4938043

[ece36568-bib-0028] Firth, J. A. , Voelkl, B. , Farine, D. R. , & Sheldon, B. C. (2015). Experimental evidence that social relationships determine individual foraging behaviour. Current Biology, 25, 3138–3143. 26585280 10.1016/j.cub.2015.09.075

[ece36568-bib-0029] Franks, D. W. , Ruxton, G. D. , & James, R. (2010). Sampling animal association networks with the gambit of the group. Behavioral Ecology and Sociobiology, 64, 493–503. 10.1007/s00265-009-0865-8

[ece36568-bib-0030] Hobson, E. A. , Avery, M. L. , & Wright, T. F. (2014). The socioecology of Monk Parakeets: Insights into parrot social complexity. The Auk: Ornithological Advances, 131(4), 756–775. 10.1642/AUK-14-14.1

[ece36568-bib-0031] Hoppitt, W. J. E. , & Farine, D. R. (2018). Association indices for quantifying social relationships: How to deal with missing observations of individuals or groups. Animal Behavior, 136, 227–238. 10.1016/j.anbehav.2017.08.029

[ece36568-bib-0032] Hoyt, J. R. , Langwig, K. E. , White, J. P. , Kaarakka, H. M. , Redell, J. A. , Kurta, A. , … Kilpatrick, A. M. (2018). Cryptic connections illuminate pathogen transmission within community networks. Nature, 563(7733), 710–713. 30455422 10.1038/s41586-018-0720-z

[ece36568-bib-0033] James, R. , Croft, D. P. , & Krause, J. (2009). Potential banana skins in animal social network analysis. Behavioral Ecology and Sociobiology, 63(7), 989–997. 10.1007/s00265-009-0742-5

[ece36568-bib-0034] Johnson, K. V. A. , Aplin, L. M. , Cole, E. F. , Farine, D. R. , Firth, J. A. , Patrick, S. C. , & Sheldon, B. C. (2017). Male great tits assort by personality during the breeding season. Animal Behavior, 128, 21–32. 10.1016/j.anbehav.2017.04.001 PMC5478380 28669996

[ece36568-bib-0035] Jones, T. B. , Evans, J. C. , & Morand‐Ferron, J. (2019). Urbanization and the temporal patterns of social networks and group foraging behaviors. Ecology and Evolution, 9(8), 4589–4602. 10.1002/ece3.5060 31031929 PMC6476747

[ece36568-bib-0036] Lloyd, K. J. , Altwegg, R. , Doutrelant, C. , & Covas, R. (2017). Factors affecting the foraging distance and duration of a colonial bird, the sociable weaver, in a semi‐arid environment. African Journal of Ecology, 56, 659–663. 10.1111/aje.12484

[ece36568-bib-0037] Maclean, G. L. (1973a). The sociable weaver, part 1: Description, distribution, dispersion and populations. Ostrich, 44, 176–190. 10.1080/00306525.1973.9639158

[ece36568-bib-0038] Maclean, G. L. (1973b). The sociable weaver, part 2: Nest architecture and social organisation. Ostrich, 44, 191–218.

[ece36568-bib-0039] Maclean, G. L. (1973c). The sociable weaver, part 5: Food, feeding and general behaviour. Ostrich, 44, 254–261. 10.1080/00306525.1973.9639162

[ece36568-bib-0040] Mourier, J. , Bass, N. C. , Guttridge, T. L. , Day, J. , & Brown, C. (2017). Does detection range matter for inferring social networks in a benthic shark using acoustic telemetry? Royal Society Open Science, 4, 170485 10.1098/rsos.170485 28989756 PMC5627096

[ece36568-bib-0041] Moyers, S. C. , Adelman, J. S. , Farine, D. R. , Moore, I. T. , & Hawley, D. M. (2018). Exploratory behavior is linked to stress physiology and social network centrality in free‐living house finches (*Haemorhous mexicanus*). Hormones and Behavior, 102, 105–113. 10.1016/j.yhbeh.2018.05.005 29758182

[ece36568-bib-0042] Newman, M. E. J. (2003). Mixing patterns in networks. Physical Review E, 67(2), 026126. 10.1103/PhysRevE.67.026126 12636767

[ece36568-bib-0043] Newman, M. E. J. (2010). Networks: An introduction. Oxford, UK: Oxford University Press.

[ece36568-bib-0044] Nomano, F. Y. , Browning, L. E. , Nakagawa, S. , Griffith, S. C. , & Russell, A. F. (2014). Validation of an automated data collection method for quantifying social networks in collective behaviours. Behavioral Ecology and Sociobiology, 68(8), 1379–1391. 10.1007/s00265-014-1757-0

[ece36568-bib-0045] Nosek, B. A. , Ebersole, C. R. , DeHaven, A. C. , & Mellor, D. T. (2018). The preregistration revolution. Proceedings of the National Academy of Sciences, 115(11), 2600–2606. 10.1073/pnas.1708274114 PMC5856500 29531091

[ece36568-bib-0046] Paquet, M. , Doutrelant, C. , Hatchwell, B. J. , Spottiswoode, C. N. , & Covas, R. (2015). Antagonistic effect of helpers on breeding male and female survival in a cooperatively breeding bird. Journal of Animal Ecology, 84, 1354–1362. 10.1111/1365-2656.12377 25850564 PMC4557059

[ece36568-bib-0047] Psorakis, I. , Voelkl, B. , Garroway, C. J. , Radersma, R. , Aplin, L. M. , Crates, R. A. , … Sheldon, B. C. (2015). Inferring social structure from temporal data. Behavioral Ecology and Sociobiology, 69, 857–866. 10.1007/s00265-015-1906-0

[ece36568-bib-0048] Rat, M. E. T. (2015). Dominance, social organisation and cooperation in the sociable weaver (*Philetairus socius*). (Doctoral dissertation). Cape Town, South Africa: University of Cape Town.

[ece36568-bib-0049] Rat, M. , van Dijk, R. E. , Covas, R. , & Doutrelant, C. (2015). Dominance hierarchies and associated signalling in a cooperative passerine. Behavioral Ecology and Sociobiology, 69, 437–448. 10.1007/s00265-014-1856-y

[ece36568-bib-0050] Silva, L. R. , Lardy, S. , Ferreira, A. C. , Rey, B. , Doutrelant, C. , & Covas, R. (2018). Females pay the oxidative cost of dominance in a highly social bird. Animal Behavior, 144, 135–146. 10.1016/j.anbehav.2018.08.006

[ece36568-bib-0051] Whitehead, H. (2008). Analyzing animal societies. Chicago, IL: Chicago University Press.

[ece36568-bib-0052] Whitehead, H. , & Dufault, S. (1999). Techniques for analyzing vertebrate social structure using identified individuals: Review and recommendations. Advances in the Study of Behavior, 28, 33–74.

[ece36568-bib-0053] Wilson, A. D. , Krause, S. , Dingemanse, N. J. , & Krause, J. (2013). Network position: A key component in the characterization of social personality types. Behavioral Ecology and Sociobiology, 67(1), 163–173. 10.1007/s00265-012-1428-y

